# Type 2 Valvular Heart Disease Affects Decision Making for Anticoagulation in Patients with Atrial Fibrillation: The UMBRIA-Fibrillazione Atriale Prospective Study

**DOI:** 10.1055/s-0039-1692202

**Published:** 2019-06-05

**Authors:** Maria Cristina Vedovati, Gianpaolo Reboldi, Giancarlo Agnelli, Paolo Verdecchia, on Behalf of the Umbria-Fibrillazione Atriale Investigators

**Affiliations:** 1 Vascular and Emergency Medicine - Stroke Unit, University of Perugia, Perugia, Italy; 2 Department of Medicine, University of Perugia, Perugia, Italy; 3 Fondazione Umbra Cuore e Ipertensione-ONLUS, Ospedale S. Maria Della Misericordia, Perugia, Italy

**Keywords:** atrial fibrillation, valvular heart disease, vitamin K antagonists, non–vitamin K oral anticoagulants

## Abstract

**Background** Valvular heart disease (VHD) and atrial fibrillation (AF) often coexist.

**Aim** We investigated whether type 2 VHD (other than moderate-severe rheumatic mitral stenosis or mechanical heart valve) influences the prescription of anticoagulants in AF.

**Methods** Umbria-Fibrillazione Atriale is a prospective multicenter registry in patients with AF. For the purpose of this study, type 2 VHD patients were propensity matched with non-VHD counterparts in a 1:1 ratio. Patients with type 1 VHD (moderate-severe mitral stenosis or mechanical heart valve) were excluded.

**Results** We identified 2,212 patients with AF and excluded 46 because data on VHD were unavailable. Type 2 VHD was present in 426 patients (19.7%). Before registry entry visit, 77.1% of type 2 VHD and 66.8% of non-VHD patients were on anticoagulants. At discharge, 90.8 and 85.2% of patients, respectively, were on anticoagulants. After propensity-score matching, 386 patient-pairs were created. In the matched sample, the likelihood of being on anticoagulants before (odds ratio [OR]: 1.43, 95% confidence interval [CI]: 1.02–2.01, *p* = 0.036) and after (1.63, 95% CI: 1.04–2.57, *p* = 0.034) the entry visit was higher in type 2 VHD than in non-VHD patients. Patients with type 2 VHD were 70% more likely to receive vitamin K antagonists (VKAs) (OR: 1.70, 95% CI: 1.28–2.27, *p* < 0.001), and 32% less likely to receive non–vitamin K oral anticoagulants (NOACs; OR: 0.68, 95% CI: 049–0.94, *p* = 0.011) than non-VHD patients.

**Conclusion** VKAs consistently outperformed NOACs as preferred treatment option in patients with type 2 VHD. This could potentially deny to these patients the well-established benefits of NOACs observed in phase III trials.

## Introduction

Valvular heart disease (VHD) and atrial fibrillation (AF) are frequent conditions which generally share an enlargement of the left atrium in addition to several risk factors such as advanced age, hypertension, diabetes, coronary heart disease, and heart failure. 1 Both AF and VHD are independent risk factors for stroke and systemic embolism. 2 VHD is associated with a higher risk of thromboembolism regardless of the underlying cardiac rhythm. 3 When AF and rheumatic mitral stenosis coexist, the thromboembolic risk is particularly high. 4 5

Management strategies for patients with AF in association with VHD have been less informed by recent randomized trials comparing vitamin K antagonists (VKAs) to non–vitamin K oral anticoagulants (NOACs). Patients with AF and moderate or severe mitral stenosis or mechanical prosthetic valves are at very high risk of thromboembolic events (∼25% per year if not anticoagulated and ∼0.8% per year if treated with anticoagulants 4 ), and therefore their random allocation to drugs different from VKAs was considered unethical. A trial specifically conducted with dabigatran versus VKAs in patients with AF and mechanical heart valves (RE-ALIGN) was prematurely interrupted because of an excess in thromboembolic and bleeding complications in the dabigatran group. 6

Definition and staging of the underlying VHD in patients with AF may affect the anticoagulation strategy. A recent Evaluated Heartvalves, Rheumatic or Artificial (EHRA) Consensus Document proposed a categorization in relation to the type of oral anticoagulant to be preferred in patents with AF and VHD. 7 The type 1 VHD refers to AF patients who require therapy with VKAs and includes patients with mitral stenosis (moderate/severe of rheumatic origin) or mechanical prosthetic valve replacement, whereas the type 2 VHD (all other types of VHD) refers to AF patients who require either VKAs or NOACs. The indication to anticoagulation in EHRA type 2 VHD should also take into consideration the thromboembolic risk according to the CHA _2_ DS _2_ -VASc score. 7

Subgroup analyses of phase III trials of NOACs in patients with AF showed that those with type 2 VHD were older, had more comorbidities including renal dysfunction, and were more frequently affected by persistent or permanent AF than patients without VHD. 7 8 Patients with type 2 VHD had higher cardioembolic and bleeding risk scores than patients without VHD. 7 8 Furthermore, irrespective of the treatment (i.e., VKAs or NOACs), type 2 VHD patients experience a worse outcome (stroke and systemic embolism, major bleeding, or all-cause death) in comparison to non-VHD patients. 7 8

In the daily practice, despite the lack of evidence from clinical trials, type 2 VHD generally is perceived as a condition of increased thromboembolic risk, thus conditioning the choice of anticoagulant treatment. This could lead to denying these patients the benefits of NOACs versus VKAs found in controlled trials. 9 10 The present study was designed to evaluate whether diagnosis of type 2 VHD influences prescription and choice of anticoagulants in patients with AF in real life.

## Methods

We included in this analysis patients enrolled in the “Umbria-Fibrillazione Atriale” study from January 2013 to December 2017. The “Umbria-Fibrillazione Atriale” study ( www.umbriafa.it ), established in 2013, is an ongoing observational registry in patients with AF. Patients are being recruited from 22 centers in Umbria, Italy. Study centers include cardiology, internal medicine, or neurology hospital units or outpatient facilities. Admission criteria include all the following: diagnosis of AF and at least one episode of AF diagnosed by electrocardiography within 1 year before the date of admission. Exclusion criteria are the presence of mechanical heart valves, moderate or severe mitral stenosis, a live expectancy less than 1 year, or refusal of informed consent. Baseline data on risk factors and treatment strategies in use before and after the registry entry visit are stored in a clinical web-based record form ( www.umbriafa.it ), with access protected by personal passwords. The patients undergo regular follow-up visits by their family doctors and/or hospital staff to ascertain their clinical status, adherence to treatment and occurrence of side effects, and major cardiovascular complications.

The informed consent is obtained from each patient and the study protocol conforms to the ethical guidelines of the 1975 Declaration of Helsinki as reflected in a priori approval by Ethical Committee and/or Institutional Review Boards of the participating centers.

This study was designed to evaluate whether diagnosis of type 2 VHD influences prescription and choice of anticoagulants in patients with AF in real life.

The following data are collected at the entry visit: age, gender, weight, height, smoke or alcohol assumption, symptoms, blood pressure, heart rate, and comorbidities. These include hypertension, congestive heart failure, diabetes, previous stroke or transient ischemic stroke or systemic embolism, vascular disease, peripheral artery disease, renal or liver disease, previous bleeding, cancer, chronic obstructive pulmonary disease, type of AF (paroxysmal, persistent, permanent), presence of pacemaker or intracardiac defibrillator, electrocardiographic and echocardiographic findings, presence of type 2 VHD, previous cardioversion/ablation, and laboratory tests (platelet number, hemoglobin level, and creatinine value). Treatments in use before and after the registry entry visit are also collected. Antithrombotic strategies are categorized as follows: none, antiplatelet agents, low-molecular-weight heparin, VKAs, or NOACs.

For the purpose of this study, congestive heart failure, renal and liver failure, and vascular diseases are defined according to the CHA _2_ DS _2_ -VASc and HAS-BLED criteria, as reported in a previous study. 11

Type 2 VHD was defined as moderate or severe mitral or aortic regurgitation, moderate or severe aortic stenosis, or mild mitral stenosis (mitral valve area > 2.0 cm ^2^ on standard echocardiography).

The primary outcome of this analysis was the use of anticoagulant treatment as reported before and at the end of the registry entry visit in type 2 VHD and in non-VHD patients. Anticoagulant treatment was defined as the use of low-molecular-weight heparin, VKAs, or NOACs.

Data analysis was performed using SAS/STAT Rel. 9.4 ( http://www.sas.com ) and R version 3 ( http://www.rproject.org ), and statistical significance was defined as a two-sided *p* -value less than 0.05. The propensity scores (PSs) for VHD status were estimated from a logistic regression model which included the following covariables: age, gender, type of AF, hypertension, diabetes, congestive heart failure, vascular disease, previous stroke or transient ischemic attack or systemic embolism, history of bleeding, severe renal failure, liver failure, and history of cancer. Type 2 VHD patients were matched in a 1:1 ratio with non-VHD patients, and balance was assessed using standardized differences with limits of −0.1 and 0.1. 12 13

Categorical data were reported as frequencies and continuous data as mean ± SD. Continuous data were compared with the use of *t* -test and categorical data were compared with the use of *χ* ^2^ test.

The likelihood of the use of anticoagulant treatment before and after the registry entry visit in type 2 VHD patients and in non-VHD patients was reported as odds ratios with 95% confidence intervals (CIs). Subgroup analyses were also set for mitral or aortic site of type 2 VHD and for type of anticoagulant (VKAs or NOACs).

## Results

**Fig. 1:**
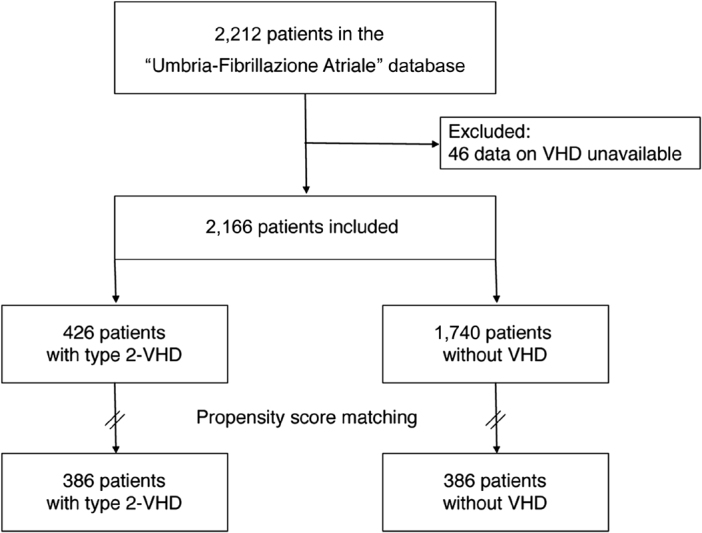
Flow diagram of the study population. VHD, valvular heart disease.

Overall, 2,212 patients with AF were evaluated and 46 were excluded because data on VHD were not available. Type 2 VHD was present in 426 (19.7%) of the 2,166 patients included in the analysis ( Fig. 1 ).

Compared with non-VHD patients, those with type 2 VHD were older, had a higher prevalence of kidney and liver disease, and had higher thrombotic and bleeding risk scores. Patients' baseline characteristics according to VHD status are shown in Table 1 .

**Table 1:** Patients' characteristics

		Overall population		*p* -Value
		Non-VHD	Type 2 VHD	
Patients, *N*		1,740	426	
Age, mean (SD)		74.9 (11)	78.2 (9)	<0.001
Gender, *N* (%)	Female	755 (43)	206 (48)	0.064
	Male	985 (56)	220 (52)	
Patterns of atrial fibrillation, *N* (%)	First diagnosed/paroxysmal	578 (33)	117 (27)	<0.001
	Persistent/permanent	1,147 (66)	309 (73)	
CHADS _2_ , mean (SD)		2.1 (1.3)	2.5 (1.3)	<0.001
CHA _2_ DS _2_ VASc, mean (SD)		3.6 (1.8)	4.3 (1.7)	<0.001
HAS-BLED, mean (SD)		1.5 (1.0)	1.7 (1.0)	<0.001
Hypertension, *N* (%)	No	351 (20)	60 (14)	0.004
	Yes	1,382 (80)	366 (86)	
Diabetes, *N* (%)	No	1,395 (81)	338 (79)	0.59
	Yes	338 (19)	88 (21)	
Congestive heart failure, *N* (%)	No	1,441 (82)	263 (62)	<0.001
	Yes	292 (17)	163 (38)	
Vascular disease, *N* (%)	No	1,335 (77)	275 (65)	<0.001
	Yes	398 (23)	151 (35)	
Previous stroke/TIA/SE, *N* (%)	No	1,411 (81)	334 (78)	0.16
	Yes	322 (19)	92 (22)	
History of bleeding, *N* (%)	No	1,662 (95)	399 (94)	0.11
	Yes	78 (4)	27 (6)	
Severe renal disease, *N* (%)	No	1,676 (97)	394 (92)	<0.001
	Yes	57 (3)	32 (7)	
Liver disease, *N* (%)	No	1,722 (99)	416 (98)	0.032
	Yes	18 (1)	10 (2)	
History of cancer, *N* (%)	No	1,573 (90)	379 (89)	0.374
	Yes	167 (10)	47 (11)	

Abbreviations: SE, systemic embolism; TIA, transient ischemic attack; VHD, valvular heart disease.

**Fig. 2:**
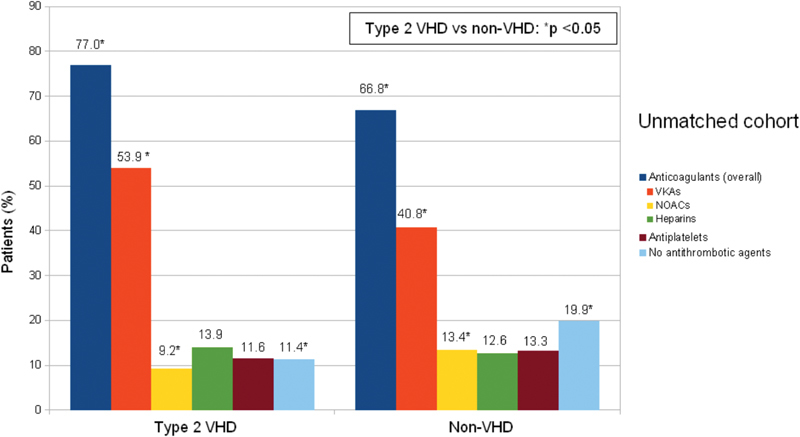
Treatment distribution in type 2 VHD and in non-VHD unmatched patients at entry visit. VHD, valvular heart disease

At registry entry visit, anticoagulant treatment was being used by 77.0 and 66.8% of type 2 VHD and non-VHD patients ( *p* < 0.001), respectively ( Fig. 2 ). VKAs were more frequently used in type 2 VHD compared with non-VHD patients: 53.9 versus 40.8% ( *p* < 0.001), while NOACs were used in 9.2% type 2 VHD and in 13.4% non-VHD patients ( *p* = 0.020). No significant differences were observed in the use of low-molecular-weight heparins (13.9% in type 2 VHD and 12.6% in non-VHD, *p* = 0.473) and of antiplatelet agents between the two groups (11.6% in type 2 VHD and 13.3% non-VHD, *p* = 0.336). The remaining 11.4 and 19.9% of type 2 VHD and non-VHD patients ( *p* < 0.001) were on no antithrombotic treatment.

At visit discharge, 90.8 and 85.2% of patients with type 2 VHD and non-VHD ( *p* = 0.002), respectively, were on anticoagulant treatment. The proportion of patients on anticoagulant treatment increased after the entry visit, but numerically less in type 2 VHD (5.6%) than in non-VHD (10.3%) patients. At visit discharge, VKAs were more frequently used in type 2 VHD compared with non-VHD patients: 54.4 versus 41.1%, respectively ( *p* < 0.001). NOACs were in use in 23.1% of type 2 VHD and in 32.4% of non-VHD patients ( *p* < 0.001). Low-molecular-weight heparins (13.4% of type 2 VHD and in 11.7% of non-VHD patients, *p* < 0.327) and antiplatelet agents (in 3.3% of type 2 VHD and in 4.8% of non-VHD, *p* = 0.171) were similarly used among the two groups. The remaining 5.9 and 10.0% of patients ( *p* = 0.009), of the two groups respectively, were on no antithrombotic treatment at visit discharge.

### Propensity Score–Matched Cohort

**Fig. 3:**
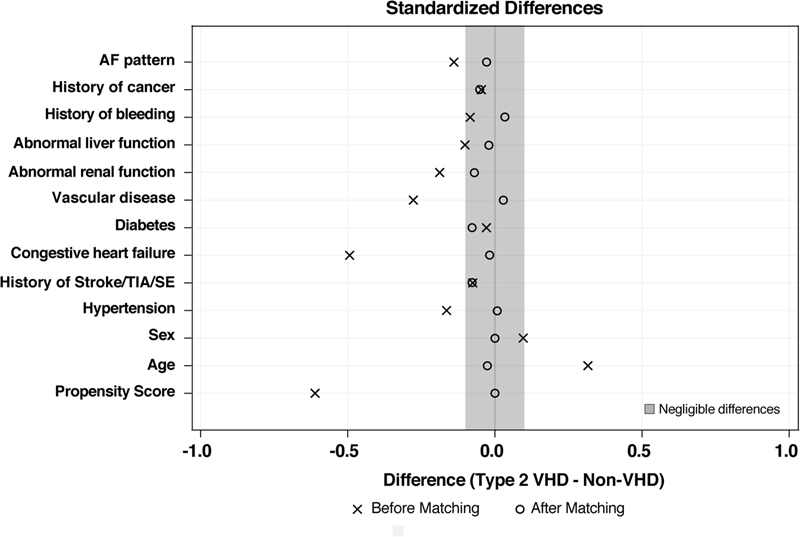
Standardized mean differences plot before and after matching. All differences for the matched observations are within the balance limits of –0.1 and 0.1 which are indicated by the shaded area. AF, atrial fibrillation; SE, systemic embolism; TIA, transient ischemic attack; VHD, valvular heart disease.

**Fig. 4:**
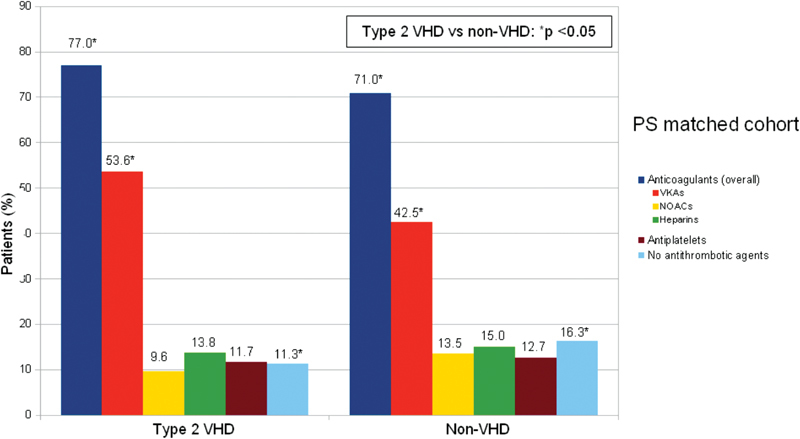
Treatment distribution in type 2 VHD and in non-VHD matched patients at entry visit. PS, propensity score; VHD, valvular heart disease.

After PS matching, 386 patient-pairs were formed. No differences were observed in patients with and without VHD after matching ( Fig. 3 ). Treatment prescription according to the presence of type 2 VHD is reported in Fig. 4 .

In the matched populations, the likelihood of anticoagulant treatment before and after the registry entry visit was consistently higher in patients with type 2 VHD than in non-VHD: 77.0 versus 71.0%; odds ratio (OR): 1.43, 95% CI: 1.02–2.01, *p* = 0.036, before and 90.9 versus 86.0%; OR: 1.63, 95% CI: 1.04–2.57, *p* = 0.034, after, respectively. Type 2 VHD patients were more likely to receive VKAs (54.7 vs. 41.2%, OR: 1.70, 95% CI: 1.28–2.27, *p* < 0.001) and less likely to receive NOACs (23.0 vs. 30.8%, OR: 0.68, 95% CI: 0.49–0.94, *p* = 0.011) when compared with non-VHD ( Table 2 ).

**Table 2:** Anticoagulants use before and after the registry entry visit in the propensity-matched sample

Treatment		At entry		At discharge	
		*N* , %	OR (95% CI, *p* -value)	*N* , %	OR (95% CI, *p* -value)
Any anticoagulant	Type 2 VHD	297, 77.0	1.43 (1.02–2.01, 0.036)	351, 90.9	1.63 (1.04–2.57, 0.034)
	Non-VHD	274, 71.0		332, 86.0	
VKAs	Type 2 VHD	207, 53.6	1.58 (1.19–2.12, 0.002)	211, 54.7	1.70 (1.28–2.27, <0.001)
	Non-VHD	164, 42.5		159, 41.2	
NOACs	Type 2 VHD	37, 9.6	0.68 (0.44–1.07, 0.093)	89, 23.0	0.68 (0.49–0.94, 0.011)
	Non-VHD	52, 13.5		119, 30.8	

Abbreviations: NOACs, non–vitamin K oral anticoagulants; VHD, valvular heart disease; VKAs, vitamin K antagonists.

### Treatment Use According to Valvular Defect

Among patients with type 2 VHD, the majority (313; 73.5%) had mitral regurgitation, while 163 (38.3%) had aortic regurgitation, 111 (26.1%) had aortic stenosis, and 28 (6.6%) had mild mitral stenosis. Before and after the referral visit, no significant differences in the use of anticoagulants were found among the various type 2 VHDs. The use of VKAs was preferred at visit discharge in patients with mild mitral stenosis compared with other type 2 VHDs, but the difference did not reach formal statistical significance (69 vs. 50%, *p* = 0.072).

One-hundred and fifty-nine patients (37.3%) had more than one valvular defect. Type 2 VHD at aortic site was present in 112 patients (26.3%), at mitral site in 181 (42.5%), and was combined in the remaining 133 patients (31.2%).

The probability to receive an anticoagulant treatment in patients with one valvular defect was similar to those with two or more defects both before the visit (77.4 vs. 76.6%, *p* = 0.854) and at discharge (92.5 vs. 91.9%, *p* = 0.837). Similarly, in patients with aortic, mitral, and combined valvular defect, no significant differences in terms of anticoagulant use were observed before the visit (77.5, 77.2, and 76.5%, respectively, *p* = 0.982) as well as at discharge (95.3, 91.2, and 91.1%, respectively, *p* = 0.395).

## Discussion

In the Umbria-Fibrillazione Atriale study, the overall proportion of patients on anticoagulants is currently nearly 90% at discharge. Besides a prevalence of anticoagulation somewhat higher than that observed in other studies, 14 the novel finding of our study is the lack of influence of type 2 VHD as determinant of the overall use of anticoagulants. We further found, however, that patients with type 2 VHD were 70% more likely to receive VKAs, and 32% less likely to receive NOACs, when compared with patients without VHD. The two groups of patients were well characterized and matched by PS, which allowed for a reliable comparison between the groups. Thus, our physicians seem to be actually more reluctant to prescribe NOACs, and more inclined to maintain or prescribe VKAs, in these patients. A possible explanation is that the presence of type 2 VHD is perceived as a condition of increased thromboembolic risk, thus conditioning the choice of anticoagulant treatment. It will be important to verify whether the recent indication of not considering VKAs as a priority option in patients with type 2 VHD 7 will impact on such attitude in the next future.

In a study from Turkey, Basaran at al addressed the issue of treatment strategies in patients with AF and VHD different from mitral stenosis or prosthetic heart valves. 15 In that study, 73.8% of patients with type 2 VHD were on oral anticoagulants, as opposed to 71.2% of patients without VHD ( *p* = 0.035). Treatment with VKAs was more prevalent in patients with type 2 VHD than in those without VHD (37 vs. 32.9%, *p* = 0.002), while NOACs were equally distributed between the two groups (37 vs. 38%, *p* = 0.324). In the study by Basaran et al, mitral regurgitation was the most prevalent type of VHD, followed by aortic regurgitation and aortic stenosis. Overall, the use of anticoagulants was less common in the study by Basaran et al than in our study. Potential explanation may be found in the differing prevalence of selected risk factors, that is, hypertension and prior stroke, among the two study populations.

The question of the choice of anticoagulant in patients with type 2 VHD remains open. As patients with moderate-severe mitral stenosis or mechanical prosthetic heart valve are at very high thromboembolic risk and have been excluded from phase III trials on NOACs compared with VKAs, these patients should continue to be treated with VKAs. However, several patients with AF and different types of VHD (i.e., moderate-severe mitral or aortic regurgitation, moderate-severe aortic stenosis) or biologic prosthetic valves have been included in these trials. For example, patients with prior valve surgery (with exclusion of mechanic valves) were excluded from RE-LY, but totalled 5.3% of the overall ROCKET-AF population, 5.2% of ARISTOTLE, and 11.5% of ENGAGE-AF. In a recent meta-analysis of these trials, the incidence of stroke or systemic embolism in patients treated with NOACs was lower than that observed in those receiving VKAs both in patients with (relative risk [RR]: 0.70, 95% CI: 0.58–0.86) and without VHD (RR: 0.84, 95% CI: 0.75–0.95). 8 Similar rates of major bleeding in patients receiving NOACs or VKAs were also observed in patients with (RR: 0.93, 95% CI: 0.68–1.27) and without VHD (RR: 0.85, 95% CI: 0.70–1.02). A significant reduction of intracranial bleedings was also observed in the NOACs group independently of VHD status (RR: 0.47, 95% CI: 0.24–0.93 and RR: 0.49, 95% CI: 0.41–059, in patients with and without VHD, respectively). 8

In a post-hoc analysis of ARISTOTLE, trial outcomes were compared according to type of VHD. 16 Overall, aortic stenosis was associated with a higher risk of stroke/systemic embolism, bleeding, and death. The efficacy and safety benefits of apixaban compared with warfarin were consistent, regardless of the presence of mitral or aortic regurgitation, and aortic stenosis.

In AF patients with bioprosthetic heart valves, the evidence is scarce. A small prospective, open-label pilot study randomized AF patients with a bioprosthetic heart valve, implanted at least 3 months before randomization, to receive dabigatran (at a dose of 110 mg twice daily) or warfarin (dose-adjusted international normalized ratio: 2–3). After 3 months of follow-up, the incidence of intracardiac thrombus detected by transesophageal echocardiography did not differ between the two groups. 17

Taken together, the results of the above trials influenced the recent American 18 and European VHD guidelines. 19 20 21 For the majority of potential clinical scenarios, there is a remarkable degree of consistency between AHA/ACC and ESC/EACTS guidelines as far as the management of patients with VHD and AF is concerned. Both societal documents consistently contend that the term “nonvalvular AF” is poorly defined and should be abandoned. Rather, the clinician should consider AF in the context of the specific VHD type and also in relation to the patient risk profile. 22

A recent consensus document endorsed by several scientific societies proposed a functional EHRA categorization in relation to the type of oral anticoagulant use in patients with AF. EHRA type 1 VHD, which refers to AF patients with “VHD needing therapy with a VKA” and EHRA type 2 VHD, which refers to AF patients with “VHD needing therapy with a VKA or a non-VKA oral anticoagulant” also taking into consideration CHA _2_ DS _2_ -VASc score risk factor components. 7 More recently, a practical guide on the use of non-VKA oral anticoagulants issued by the European Heart Rhythm Association, in patients with biological valves or after valve repair, stated that “the use of a NOAC for the management of concomitant AF is considered to be a valid option.” However, in patients with biological mitral prosthesis implanted for rheumatic mitral stenosis and large and severely diseased atria, VKAs may still remain a valid option, although more data are needed. 21

Our study was conducted in patients with EHRA type 2 VHD, for whom no clear preference emerged between VKAs and NOACs in clinical trials. During the study period, there was not enough data regarding the safety and efficacy profile of NOACs or VKAs in AF and type 2 VHD. However, we found that the presence of VHD clearly affected decision making for anticoagulation in these patients with AF. The likelihood of anticoagulant treatment before and after the registry entry visit was consistently higher in patients with type 2 VHD than in those without VHD; moreover, type 2 VHD patients were more likely to receive VKAs when compared with patients without VHD.

The present study has several limitations that are quite common in the analysis of observational studies. First, similarly to other observational studies, the risk of selection bias in the treatment allocation to VKAs or NOACs is inherent and cannot be excluded. To overcome this limitation, we propensity matched patients with type 2 VHD with those without VHD. Second, the present analysis was cross-sectional, and not intended to investigate the long-term risk of thromboembolic and bleeding complications in relation to the choice of anticoagulant in the two groups. At last, no bioprosthetic valves (including TAVI) or previous valve repairs were reported in the included population.

## Conclusion

A notable finding of our study was the large proportion of patients on anticoagulants, which exceeded 90%, regardless of the presence of type 2 VHD. However, VKAs remained the preferred treatment option in patients with type 2 VHD. Controlled trials between VKAs and NOACs, properly stratified by type of VHD, are definitely needed to support evidence-based clinical decisions in this special patient population.

## Notes

Appendix A: Umbria-Fibrillazione Atriale Study
**Steering Committee** : Giancarlo Agnelli; Giuseppe Ambrosio; Fabio Angeli; Claudio Cavallini; Adriano Murrone; Gianpaolo Reboldi; Paolo Verdecchia (Chairperson); Gianluca Zingarini.

**No-profit Sponsor** : Fondazione Umbra Cuore e Ipertensione—ONLUS.

**Scientific Secretary** : Fabio Angeli.

**Participating Investigators** : Hospital of Ancona and University of Ancona, Department of Cardiology (A. Capucci, F. Guerra, G. Ciliberti); Hospital of Ascoli Piceno, Department of Cardiology (L. Moretti, P. Marchese, F. Gennaro, G. Mazzotta); Hospital of Fabriano, Department of Cardiology (S. Coiro, M. Politano, P. Scipione); Hospital of Amelia, Department of Cardiology (M.L. Suadoni, S. Bergonzini); Hospital of Assisi, Department of Medicine (P. Verdecchia, G. Molini, A. Aita); Hospital of Branca, Department of Medicine (S. Radicchia, O. Cazzato); Hospital of Branca, Department of Cardiology (E. Capponi, D. Cosmi, G. Mazzotta); Hospital of Branca, Department of Neurology (D. Giannandrea); Hospital of Città di Castello, Department of Neurology (S. Ricci, M.R. Condurso, L. Greco); Hospital of Città di Castello, Department of Cardiology (A. Murrone, A. Contine, L. Marinacci, K. Mboumi); Hospital of Castiglione del Lago (C. Dembech, N. Sacchi, M. Guerrieri, M. Martinelli); Hospital of Perugia and University of Perugia, Medicina Interna e Vascolare (G. Agnelli, M.G. De Natale, C. Becattini, M.C. Vedovati); Hospital of Perugia and University of Perugia, Cardiologia e Fisiopatologia Cardiovascolare (G. Ambrosio, F. Angeli, D. Turturiello, F. Scavelli, M. Reccia, G. Giuffrè); Hospital of Perugia and University of Perugia, Medicina Interna (M. Pirro, V. Bianconi, A. Labate); Hospital of Perugia, Struttura Complessa di Cardiologia (C. Cavallini, G.L. Zingarini, F. Notaristefano, C. Riccini); Hospital of Perugia, Struttura Complessa di Pronto Soccorso (P. Groff, V. Mommi); Hospital of Foligno, Struttura Complessa di Cardiologia (G. Bagliani, C. Andreoli, C. Mangialasche); Hospital of Orvieto, Struttura Complessa di Cardiologia (R. Di Cristofaro); USL Umbria 1, Cardiologia Ambulatoriale 1 (M.G. Pinzagli); USL Umbria 2, Cardiologia Ambulatoriale 2 (L. Filippucci, A. Faleburle); USL Umbria 2, Cardiologia Ambulatoriale (S. Repaci), USL Umbria 2, Cardiologia Ambulatoriale (G. Proietti); Hospital Media Valle del Tevere, Struttura Complessa di Medicina (U. Paliani, C. Fuoco, M.G. Conti, A. Cardona, C. Bartolini); Hospital of Narni, Struttura Complessa di Medicina (P. Rinaldi), Hospital of Terni, Struttura Complessa di Cardiologia (G. Carreras, E. Boschetti, C. Poltronieri, A. Crocetti, G. Tilocca, G. Khoury); Hospital of Terni, Medicina Interna (G. Vaudo, G. Pucci, L. Sanesi, R. Sgariglia, S. Alessio, A. Cerasari, I. Dominioni).
